# Gestational age at birth and risk of intellectual disability without a common genetic cause

**DOI:** 10.1007/s10654-017-0340-1

**Published:** 2017-12-06

**Authors:** Hein Heuvelman, Kathryn Abel, Susanne Wicks, Renee Gardner, Edward Johnstone, Brian Lee, Cecilia Magnusson, Christina Dalman, Dheeraj Rai

**Affiliations:** 1 Centre for Academic Mental Health, Population Health Sciences, Bristol Medical School, University of Bristol, Oakfield House, Oakfield Grove, Bristol, BS8 2BN UK; 2 Centre for Women’s Mental Health, Manchester Academic Health Sciences Centre, Institute of Brain Behaviour and Mental Health, University of Manchester, 3rd Floor Jean McFarlane Building, Oxford Road, Manchester, M13 9PL UK; 3 Manchester Mental Health and Social Care Trust, Chorlton House, 70 Manchester Road, Manchester, M21 9UN UK; 4 Department of Public Health Sciences, Karolinska Institutet, 171 77 Stockholm, Sweden; 5 Centre for Epidemiology and Community Medicine, Stockholm County Council, 171 29 Solna, Sweden; 6 Maternal and Fetal Health Research Centre, Manchester Academic Health Sciences Centre, Institute for Human Development, University of Manchester, St Mary’s Hospital, Oxford Road, Manchester, M13 0WL UK; 7 Department of Epidemiology and Biostatistics, A.J. Drexel Autism Institute, Drexel University School of Public Health, Philadelphia, PA USA; 8 Avon & Wiltshire Mental Health Partnership NHS Trust, Bristol, UK

**Keywords:** Intellectual disability, Gestational age, Stockholm Youth Cohort, Regression splines, Siblings, Post-term birth

## Abstract

Preterm birth is linked to intellectual disability and there is evidence to suggest post-term birth may also incur risk. However, these associations have not yet been investigated in the absence of common genetic causes of intellectual disability, where risk associated with late delivery may be preventable. We therefore aimed to examine risk of intellectual disability without a common genetic cause across the entire range of gestation, using a matched-sibling design to account for unmeasured confounding by shared familial factors. We conducted a population-based retrospective study using data from the Stockholm Youth Cohort (n = 499,621) and examined associations in a nested cohort of matched outcome-discordant siblings (n = 8034). Risk of intellectual disability was greatest among those born extremely early (adjusted OR_24 weeks_ = 14.54 [95% CI 11.46–18.44]), lessening with advancing gestational age toward term (aOR_32 weeks_ = 3.59 [3.22–4.01]; aOR_37__weeks_ = 1.50 [1.38–1.63]); aOR_38 weeks_ = 1.26 [1.16–1.37]; aOR_39_ weeks = 1.10 [1.04–1.17]) and increasing with advancing gestational age post-term (aOR_42 weeks_ = 1.16 [1.08–1.25]; aOR_43 weeks_ = 1.41 [1.21–1.64]; aOR_44 weeks_ = 1.71 [1.34–2.18]; aOR_45 weeks_ = 2.07 [1.47–2.92]). Associations persisted in a cohort of matched siblings suggesting they were robust against confounding by shared familial traits. Risk of intellectual disability was greatest among children showing evidence of fetal growth restriction, especially when birth occurred before or after term. Birth at non-optimal gestational duration may be linked causally with greater risk of intellectual disability. The mechanisms underlying these associations need to be elucidated as they are relevant to clinical practice concerning elective delivery around term and mitigation of risk in post-term children.

**Electronic supplementary material:**

The online version of this article (10.1007/s10654-017-0340-1) contains supplementary material, which is available to authorized users.

## Introduction

Intellectual disability is a group of developmental disorders evident early in childhood and characterized by cognitive and functional impairments as a result of delayed or incomplete development of the mind [1]. Individuals with intellectual disability have a reduced ability to understand new or complex information and to learn and apply new skills, resulting in a reduced ability to cope independently [2]. Intellectual disability is thought to affect over 1% of the population [3, 4] although estimates vary with the demographic and socioeconomic composition of study populations [4, 5] and with definitions and study design [5, 6]. The cost of intellectual disability to individuals and society is substantial [7] and people living with these disabilities often face significant stigma [8] while encountering substantial health and social inequalities and early mortality [9].

Although there are many risk factors, a specific cause is identified for less than half of those with mild disabilities (IQ range 50–69) who make up the majority of cases [3, 10]. Mild intellectual disability often clusters within families [10] suggesting that genetic or other shared familial factors may influence risk. When disabilities are more severe, specific causes are identified in over 75% of cases, often involving genetic or chromosomal abnormalities and inborn errors of metabolism [10]. When intellectual disability is present without a specific genetic or chromosomal cause, it is associated with advanced maternal age, maternal risk behaviors or medical problems during pregnancy and fetal growth restriction [11], suggesting that these may be risk factors.

While it is known that children born preterm (< 37 completed weeks) are at greater risk of intellectual disability than those born at term [12], less is known about the development of risk along the gestational course, or about risk among post-term children (> 41 weeks). An early smaller study found no difference in intelligence between children born at term or post-term, although it was limited in terms of statistical power and length of follow-up [13]. More recent, larger, studies have suggested post-term birth may be associated with a range of adverse neurological, developmental, behavioural and emotional outcomes in early childhood [14, 15] and there is increasing evidence to suggest it is associated with cognitive and academic deficits in later childhood and adolescence [16–19], especially when the baby is growth-restricted [18].

The association between the full range of gestational duration, from very early to very late births, and intellectual disability has not yet been examined in population-based studies. Furthermore, the evidence to date is insufficient because of incomplete control of confounding from shared familial factors and insufficient recognition that genetic causes of intellectual disability may also influence gestational duration [20, 21].

Therefore, in a large Swedish population-based cohort, we aimed to: (1) examine the associations between gestational age and intellectual disability without a common genetic cause, taking into account a range of potential confounders; (2) examine interactions between gestational duration and fetal growth in relation to risk of intellectual disability; and (3) explore the causal nature of associations between gestational duration and risk of intellectual disability in a nested cohort of matched outcome-discordant siblings.

## Methods

### Study cohort

The Stockholm Youth Cohort is a register-based cohort of all individuals who lived in Stockholm County for at least 1 year between 2001 and 2011 and were aged between 0 and 17 years during that period (n = 736,180) [22]. Using unique personal identification numbers, cohort members and their first-degree relatives were linked with a range of national and regional registers including information on pregnancy- and birth related characteristics, socioeconomic characteristics and medical and psychiatric diagnoses.

**Fig. 1:**
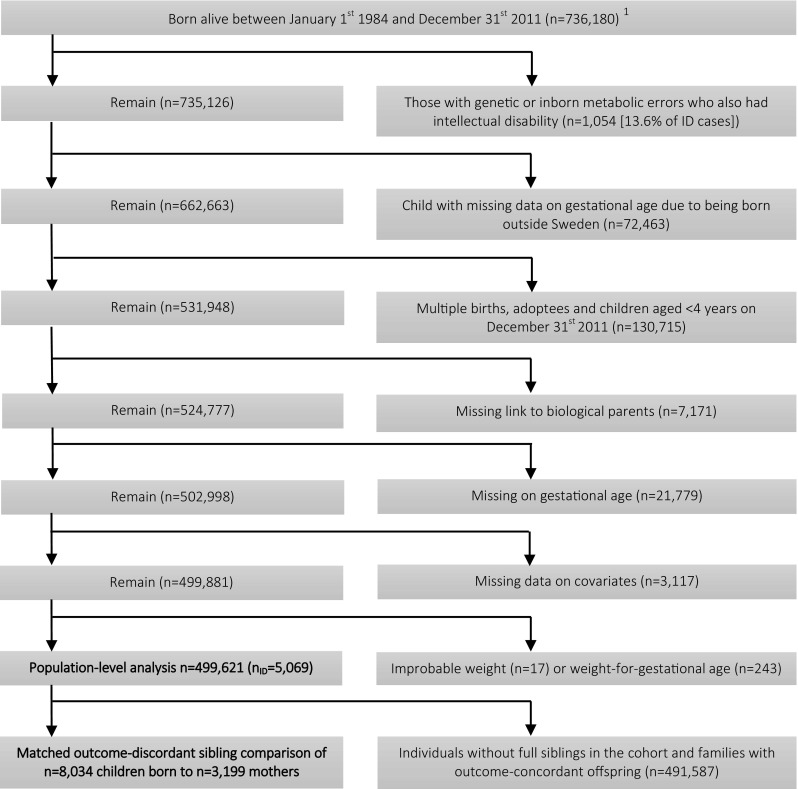
Selection of the study cohort

We excluded individuals with genetic and inborn metabolic syndromes who had been diagnosed with intellectual disability (13.6% of cases in our study population), children born outside Sweden, multiple births, adoptees, children < 4 years of age by the end of follow up on the 31st of December 2011, with a missing link to biological parents, or with missing data on gestational age or other covariates (Fig. 1). To account for potential recording errors, we excluded individuals with improbable birth weights (< 350 g or > 6000 g) and those with improbable combinations of birth weight and gestational age by deleting observations with values smaller than the 25th percentile minus 3 interquartile ranges, or larger than the 75th percentile plus 3 interquartile ranges, from sex- and week-specific birth weight distributions (Table S8) [18]. This left a cohort of 499,621 individuals to examine population-level associations between gestational age and intellectual disability. To examine associations among matched siblings, we excluded individuals without full siblings in the cohort and families with outcome-concordant offspring (n = 491,587) leaving a cohort of 8034 matched outcome-discordant full siblings.

### Exposure

We obtained information on gestational age at birth from the Medical Birth Register (MBR), constructing a categorical variable to define extremely to very preterm births (21–31 completed weeks), moderately to late preterm births (32–36 weeks), term births (37–41 weeks), post-term births (42 weeks) and very post-term births (43–45 weeks) for use in descriptive statistics and as an exposure variable in regression analyses. We also used a continuous definition of gestational age (in days) for regression analyses.

### Outcome

We used a multisource ascertainment approach to identify cohort members with intellectual disability, similar to the case identification for autism described elsewhere [22]: We used the national patient register, the Stockholm county child and adolescent mental health register, the Stockholm County healthcare database (VAL) and the Stockholm adult psychiatric register to identify all inpatient or outpatient diagnoses of intellectual disability recorded using ICD-10 (F70-79) and DSM-IV (317-318) codes and supplemented these diagnoses with a record of care at specialist habilitation services for individuals with intellectual disability in Stockholm County. We identified individuals with genetic defects and inborn errors of metabolism commonly associated with intellectual disability to identify cases where a known genetic or metabolic cause was present (Table S1).

### Covariates

To control for secular change in obstetric and diagnostic practice, we obtained year of birth from the Medical Birth Register (MBR). We then identified additional covariates which in the literature have been associated with pregnancy duration and risk of intellectual disability in offspring. From the MBR, we extracted data for offspring sex [5, 23], parity (1/2/3/4 +) [11, 24], maternal age (< 20/20–24/25–29/30–34/35–39/40–44/45 +) [11, 24], gestational diabetes [11, 25] and gestational hypertension or preeclampsia [11, 26]. We obtained birth weights [11] from the MBR to construct a measure of weight-for-gestational age by examining week- and sex-specific birth weight distributions, and identifying those in the lower and upper deciles of these distributions as born small or large for gestational age respectively. Weight-for-gestational age is therefore conceptualized as the distance between the birth weight of an individual and the average birth weight of all who were born in the same gestational week as that individual. As weight-for-gestational age is orthogonal to gestational age itself, this avoids the issue of collinearity when the measure is included as a covariate in the analytical model. To examine potential interactions between gestational age and weight-for-gestational age, we constructed a categorical measure to identify those born preterm (< 37 weeks) and small for gestational age, appropriate for gestational age (11th centile to 90th centile) or large for gestational age; those born at term (37–41 weeks) and small, appropriate or large for gestational age; and those born post-term (≥ 42) and small, appropriate or large for gestational age. We also extracted information for maternal and paternal country of birth (Sweden/other Nordic/other European/Russia or Baltic States/Africa/Middle East/Asia or Oceania/North America/South America) [5, 27], maternal and paternal history of psychiatric treatment [28, 29], quintiles of disposable family income adjusted for inflation and family size [30, 31], and parental educational attainment (≤ 9 years/10–12 years/≥ 13 years) [31, 32] at (or as close as possible to) birth.

### Statistical analyses

Analyses were conducted in Stata/MP version 14.2. We examined the characteristics of the study cohort by gestational duration at birth. To examine population-level associations between gestational duration and risk of intellectual disability, we used generalized estimating equation (GEE) multivariable regression models with a logit link function, exchangeable correlation structure and robust variance estimators to ensure that the standard errors of our estimates were robust against clustering of intellectual disability within families [33]. We calculated restricted cubic regression splines based on five knot locations (5th, 27th, 50th, 73rd and 95th percentiles of the gestational age distribution) to allow for non-linear associations between continuously varying gestational duration and later risk of intellectual disability [34]. We statistically adjusted our estimates for covariates and calculated odds ratios by continuously varying gestational age at birth to estimate risk of intellectual disability associated with birth at specific moments along the gestational course. We investigated potential interactions between gestational age and fetal growth using GEE models with a categorical exposure variable to assess risk of intellectual disability among those born at varying gestational duration (preterm/term/post-term) and weight for gestational age (small/appropriate/large) with statistical adjustment for confounders.

In a nested cohort of matched outcome-discordant siblings we examined associations between continuously varying gestational age and risk of intellectual disability with conditional likelihood logistic regression models. This allowed us to explore the potential influence of unobserved familial traits, e.g. residual genetic risk/unmeasured socioeconomic factors/parental health behaviors, which may have confounded associations between gestational length and risk of intellectual disability. If we were to observe associations at the population level, non-association within families would suggest confounding by these shared familial traits. Conversely, replication of population-level associations within families would suggest they were robust against shared familial confounding, thereby allowing stronger causal inference from our result [35]. We statistically adjusted within-family associations for non-shared confounding characteristics including sex, parity, gestational diabetes, hypertension or preeclampsia, weight for gestational age, maternal and paternal age, disposable family income quintile, and parental educational attainment.

### Sensitivity analyses

We compared characteristics for those with missing and complete data to assess whether our estimates may have been affected by selection bias (Table S2). To ensure that the association between gestational age and intellectual disability was not driven by presence of co-occurring autism spectrum disorder or attention deficit hyperactivity disorder (which are associated with intellectual disability [36–38] and for which risk may also vary by gestational age [39, 40]) we examined associations in a subset of the cohort without a record of these conditions (Figure S1 and Table S3). We examined whether the risks of intellectual disability associated with preterm or post-term birth varied with mode of delivery (Tables S4 and S5) using categorical measures to identify those born vaginally or by Caesarean section and in unassisted or forceps-/ventouse-assisted deliveries at varying gestational duration. Finally, we conducted post hoc analyses to assess whether risk varied among children born in spontaneous or induced deliveries at varying gestational duration (Table S6).

## Results

Prevalence of intellectual disability without a common genetic cause was estimated at 1% in our study population (Fig. 1). Characteristics of the study cohort are described in Table 1. Prevalence among those born at term gestation was 0.9%. By contrast, 5.6% of children born extremely to very preterm and 1.6% of those born very post-term had intellectual disability.

**Table 1:** Characteristics of the sample by exposure status

	Extremely to very preterm	Moderate to late preterm	Term	Post-term	Very Post-term
Gestational weeks	21–31	32–36	37–41	42	43–45
Number of observations	2601	20,271	438,215	34,828	3706
Percentage of the cohort	0.5	4.1	87.7	7.0	0.7
	%	%	%	%	%
Female child	45.2	46.3	49.3	44.1	43.9
Mother’s number of prior pregnancies					
0	53.4	53.4	44.2	53.8	61.5
1	27.1	28.4	37.0	30.1	23.5
2	12.5	11.8	13.6	11.6	10.0
3 +	7.0	6.4	5.3	4.5	5.0
Birth weight in grams					
< 2500	99.7	37.6	1.1	0.1	0.1
2500–4500	0.4	62.5	98.6	98.5	98.0
> 4500	0.0	0.0	0.3	1.4	1.9
Gestational hypertension or preeclampsia	23.6	12.9	3.2	1.9	1.5
Gestational diabetes	1.5	1.7	0.8	0.4	0.4
Delivery by Caesarean Section	58.9	32.4	13.3	16.5	22.5
Delivery assisted with ventouse or forceps	1.4	4.7	8.0	14.1	14.9
Maternal psychiatric history	38.6	37.0	32.7	31.5	33.0
Paternal psychiatric history	23.7	22.6	20.9	20.4	21.2
Family disposable income quintile at birth					
Lowest	14.3	15.1	14.7	13.3	15.4
Second	21.7	20.4	20.8	19.4	19.1
Third	19.9	20.4	21.6	21.1	19.3
Fourth	22.3	22.3	21.6	22.6	22.1
Highest	21.8	21.8	21.4	23.6	24.2
Parental educational attainment at birth					
≤ 9 years	9.0	8.0	6.5	5.8	7.0
10–11 years	42.0	43.3	40.5	39.9	40.7
≥ 13 years	49.0	48.7	53.0	54.2	52.3
Maternal age					
≤ 20	1.8	2.6	1.8	1.6	1.9
20–24	13.0	15.7	14.7	13.4	16.3
25–29	26.6	30.3	31.0	31.3	32.0
30–34	32.1	31.1	33.8	34.9	31.8
35–39	21.4	16.4	15.7	16.0	15.1
40 +	5.2	4.0	3.2	2.8	2.9
Paternal age					
≤ 20	0.7	0.8	0.5	0.5	0.4
20–24	7.2	8.9	7.2	7.0	8.2
25–29	20.8	24.2	23.5	23.5	25.0
30–34	30.8	31.5	33.5	33.7	32.5
35–39	22.8	20.4	22.0	22.0	21.4
40 +	17.7	14.2	13.3	13.3	12.6
Maternal country of birth					
Sweden	72.2	75.2	76.0	78.5	78.5
Other Nordic	5.1	4.6	4.2	4.2	4.6
Other European	4.3	3.6	3.6	3.5	3.3
Baltic States /Russia	0.6	0.5	0.5	0.7	0.5
Africa	5.3	3.1	3.4	4.9	5.5
Middle East	7.3	6.7	7.0	4.8	4.5
Asia /Oceania	3.2	3.7	2.8	1.7	1.7
North America	0.5	0.6	0.6	0.6	0.4
South America	1.5	2.0	1.9	1.3	1.1
Paternal country of birth					
Sweden	70.7	74.7	74.7	77.3	77.6
Other Nordic	4.3	3.5	3.2	3.0	3.4
Other European	4.6	4.5	4.7	4.6	4.5
Baltic States /Russia	0.2	0.2	0.2	0.2	0.2
Africa	6.2	4.1	4.1	5.7	5.8
Middle East	8.6	7.6	8.3	5.8	5.5
Asia/Oceania	2.4	2.3	1.9	1.2	1.1
North America	0.8	0.7	0.8	0.7	0.70
South America	2.3	2.3	2.2	1.5	1.21
Intellectual disability	5.6	1.8	0.9	1.0	1.6

**Fig. 2:**
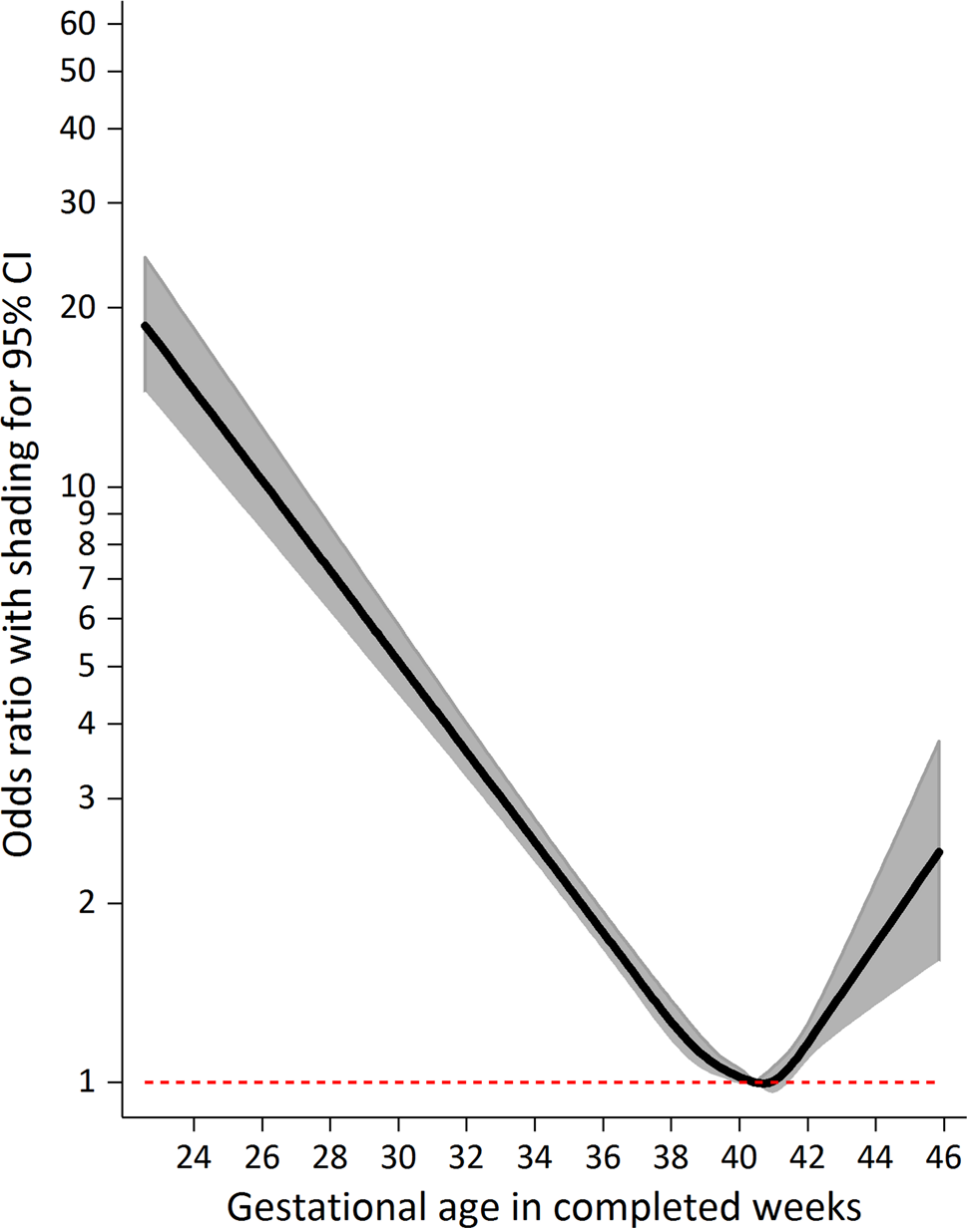
Population-level association between gestational duration and risk of intellectual disability. Notes: The population-level association (N = 499,621) was estimated using a generalized estimating equations model with a logit link, and adjusted statistically for year of birth, child sex, parity, gestational hypertension or preeclampsia, gestational diabetes, birth weight for gestational age, maternal and paternal age, maternal and paternal psychiatric history, maternal and paternal country of birth, family disposable income quintile at birth, and parental educational attainment at birth. Those born at 40 weeks and 3 gestational days are the referent

Examining associations between gestational duration and risk of intellectual disability in a model using a continuous exposure variable with statistical adjustment for potential confounders (Fig. 2), the adjusted odds ratio (aOR) for risk at extremely preterm birth (at 24 weeks) was estimated at 14.54 [95% CI 11.46–18.44]. This risk decreased with gestational age towards term (aOR_32 weeks_ = 3.59 [3.22–4.01]; aOR_37 weeks_ = 1.50 [1.38–1.63]; aOR_38 weeks_ = 1.26 [1.16–1.37]; aOR_39 weeks_ = 1.10 [1.04–1.17]) after which it increased with gestational age post-term (aOR_42 weeks_ = 1.16 [1.08–1.25]; aOR_43 weeks_ = 1.41 [1.21–1.64]; aOR_44 weeks_ = 1.71 [1.34–2.18]; aOR_45 weeks_ = 2.07 [1.47–2.92]).

We report associations using a categorical exposure variable in an online supplement (Table S7). Irrespective of gestational length, risk of intellectual disability was greatest among those showing evidence of fetal growth restriction (Table 2).

**Table 2:** Interaction between gestational duration and fetal growth in relation to risk of intellectual disability

Gestational duration^a^/weight-for-gestational age category^b^	Odds ratio^c^	95% CI		*p*	n (%)^d^	N^e^	Percentage of the cohort (%)
		Lower	Upper				
Preterm/small	3.77	2.97	4.84	< 0.001	73 (3.8)	1935	0.4
Preterm/appropriate	2.24	2.01	2.51	< 0.001	380 (2.1)	18,256	3.7
Preterm/large	2.36	1.81	3.07	< 0.001	61 (2.3)	2681	0.5
Term/small	1.88	1.73	2.05	< 0.001	731 (1.8)	41,579	8.3
Term/appropriate	1.00				3008 (0.9)	352,016	70.5
Term/large	1.06	0.95	1.18	0.27	402 (0.9)	44,620	8.9
Post-term/small	2.29	1.83	2.85	< 0.001	85 (2.2)	3822	0.8
Post-term/appropriate	1.11	0.98	1.25	0.10	292 (1.0)	30,840	6.2
Post-term/large	1.22	0.87	1.69	0.24	37 (1.0)	3872	0.8

^a^Preterm was defined as birth < 37 completed weeks of gestation. Term birth was defined as birth between 37 and 41 completed weeks of gestation. Post-term birth was defined as birth at ≥ 42 completed weeks of gestation

^b^Fetal growth categories were defined as small-for-gestational age [in the lowest decile of the gestational age-specific birthweight distribution], appropriate-for-gestational age [in the 11th to 90th decile of the gestational age-specific birthweight distribution] and large-for-gestational age [in the upper decile of the gestational age-specific birthweight distribution]

^c^Population-level associations were estimated using a generalized estimating equations model with a logit link, and adjusted statistically for year of birth, child sex, parity, gestational hypertension or preeclampsia, gestational diabetes, maternal and paternal age, maternal and paternal psychiatric history, maternal and paternal country of birth, family disposable income quintile at birth, and parental educational attainment at birth

^d^Number and percentage of ID cases within gestational duration/fetal growth category

^e^Number of observations within gestational duration/fetal growth category

^f^N = 499,621

**Fig. 3:**
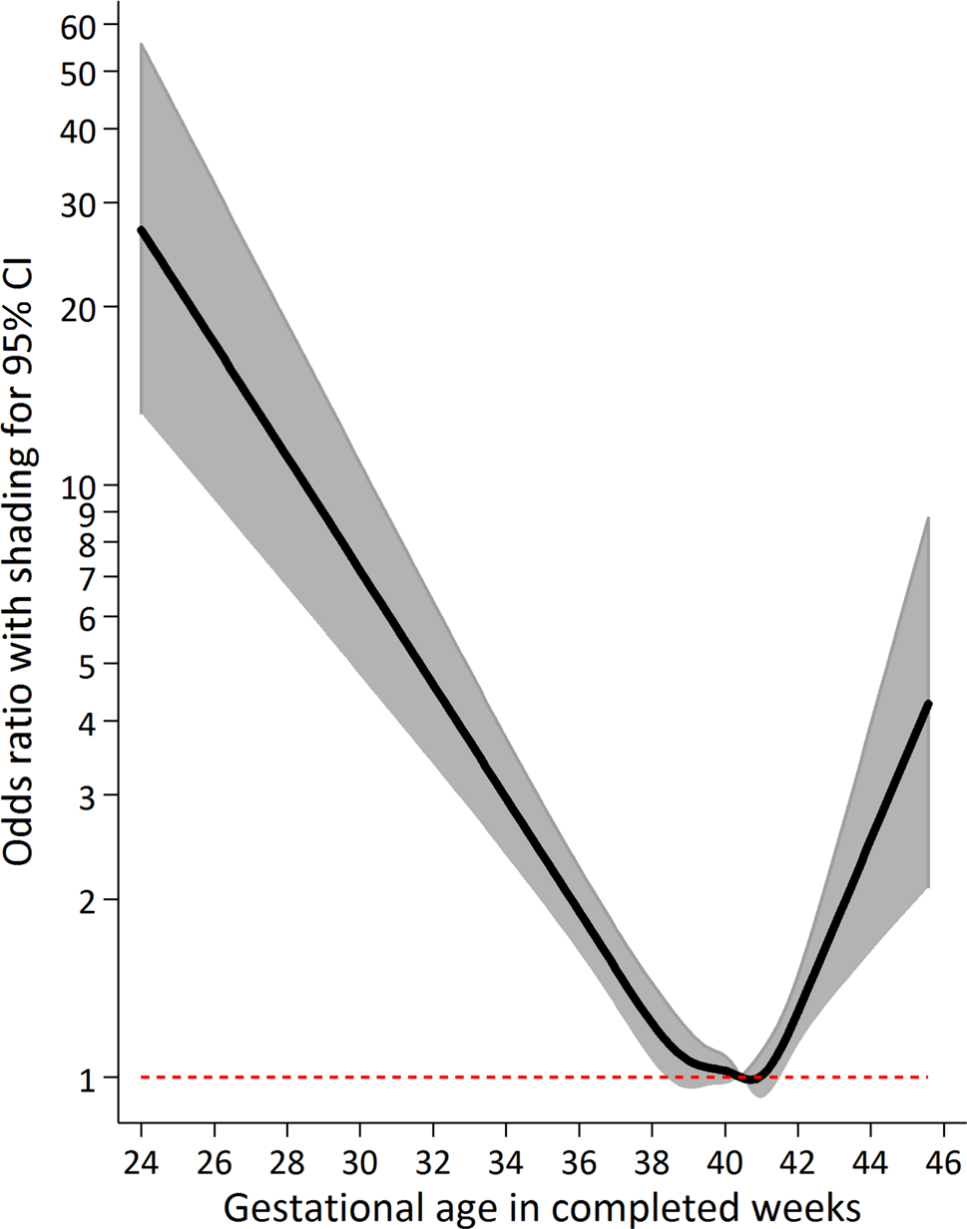
Within-family association between gestational duration and risk of intellectual disability. Notes: The within-family association (N = 8034) was estimated using a conditional likelihood logistic regression model, and adjusted statistically for year of birth, child sex, parity, gestational hypertension or preeclampsia, gestational diabetes, birth weight for gestational age, maternal and paternal age, family disposable income quintile at birth, and parental educational attainment at birth. Those born at 40 weeks and 3 gestational days are the referent.

This difference was most pronounced in the preterm group, but our results suggest risk of intellectual disability was also increased among children born post-term and growth-restricted. Associations between gestational length and risk of intellectual disability persisted when we repeated our analysis in a nested cohort of outcome-discordant siblings (Fig. 3, Table S7).

In a subset of the cohort without a diagnosis of ASD or ADHD, pre- and post-term birth remained associated with increased risk of intellectual disability (Figure S1, Table S3). Among those born at 21–31 completed weeks of gestation, risk of intellectual disability was lesser when the baby was delivered by Caesarean section, while Caesarean birth was associated with greater risk than vaginal birth between 37 and 41 weeks gestation (Table S4). There was no consistent variation in risk due to unassisted versus assisted delivery within gestational age categories (Table S5). Importantly, risk of intellectual disability associated with early or late birth remained when considering those born in vaginal or unassisted deliveries (Tables S4 and S5). Among those born between 37 and 41 weeks, risk of intellectual disability was greater when birth was induced (Table S6). This effect existed independently of the influence of fetal growth restriction or other potential confounders. Finally, children born in induced post-term deliveries were at greater risk of intellectual disability than children born spontaneously at term, while the increase in risk associated with spontaneous post-term birth was lesser (Table S6).

## Discussion

In this large population-based study, we found a greater risk of intellectual disability without a common genetic cause among preterm and post-term births compared with term births. Risk also varied within the term period and was lowest when the child was born at 40–41 completed weeks’ gestation. These associations were evident in analyses using the full sample, as well as in a nested cohort of matched outcome-discordant siblings. Risk of intellectual disability was greatest among those showing evidence of fetal growth restriction, especially when born before or after term. To our knowledge, this is the first total-population study to estimate risk of intellectual disability without a common genetic cause over the entire range of gestation using high-quality prospectively measured data. In addition to a range of measured confounders, this study explored the influence of unmeasured familial effects using a matched sibling design. This allowed us to take into account unmeasured familial confounding of the association between intellectual disability and gestational length, as these traits are heritable within families [10, 41, 42].

There were several limitations. First, 5% of the study cohort had missing data on gestational age at birth or other covariates. Although we cannot know with certainty how these exclusions may have affected our result, sensitivity analyses suggest that our estimates may have been conservative as we may have excluded preterm children with higher prevalence of intellectual disability (Table S2). Second, we did not have information on whether gestational length was calculated by the mother’s report of her last menstrual period or based on ultrasound measurement in specific pregnancies. As our sample includes births from 1984 onwards, it is likely that there is greater measurement error in earlier cohort years, where gestational length would have been estimated by last menstrual period for a larger proportion of pregnancies. This may have resulted in overestimation of rates of post-term birth [43–45] and underestimation of population-level [46] and within-family associations [47] between gestational length and later risk of intellectual disability. Third, while the matched-sibling design provides a powerful method to examine the influence of shared confounding, it is more sensitive than traditional methods to confounders not perfectly shared by the siblings. Selection based on exposure-discordance could also prompt discordance in terms of non-shared confounding characteristics, which may bias the within-family effect [47]. The size and direction of such bias depends on the similarity or dissimilarity of matched siblings in terms of exposure and confounding characteristics [47]. Given that measurement error in the gestational age variable would have downwardly biased our estimate of the within-family effect, additional bias due to sibling non-shared confounding would have either offset this downward bias or further enhanced it. Fourth, there may be bias due to omitted non-shared confounding characteristics in our matched sibling analyses. For example, it is possible that prenatal infection [48], maternal obesity [49, 50], or use of drugs or alcohol [51] may have influenced gestational length and resulted in greater risk of offspring intellectual disability in as far as these factors were present in one pregnancy but not the other.

The mechanisms underlying our findings are likely to differ depending on whether birth occurred before or after the due date. With regards to preterm birth, perturbations in development of the fetal brain because of shortened gestation can increase risk for longer-term neurodevelopmental problems [52, 53]. Our findings for preterm small-for-gestational age children would suggest that these effects might become particularly apparent if the fetus is already growth-restricted. After birth, further injury to the brain could result from respiratory support for preterm infants with immature pulmonary function [54]. Mechanisms linking post-term birth with later risk of intellectual disability might involve placental deterioration or insufficiency causing fetal hypoxia or nutritional deficiencies [55], which in turn could result in injury to the fetal brain. Meconium aspiration, which is more common in post-term birth [55], may result in neonatal asphyxia thereby incurring risk for brain injury and later neurodevelopmental problems [56].

Our finding of associations among those born in unassisted or vaginal deliveries suggested that adverse obstetric circumstances did not explain the higher risk of intellectual disability associated with birth at < 37 or > 42 weeks. Furthermore, our findings suggest that risk of intellectual disability increases with induction of labor at further post-term gestation, although these estimates are likely to be biased by the higher risk nature of induced pregnancies as a whole (Table S6). Risk of intellectual disability may have also increased with advancing post-term gestational age when delivery started spontaneously, although our data may have been underpowered to detect these subtler effects (Table S6). Importantly, due to the observational nature of our study, and given that the decision to induce labor will be informed by other factors than gestational length alone, we cannot infer from our data whether the risks associated with post-term delivery could be curtailed by induction of labor around term. This question will therefore be better answered by randomized studies designed specifically for the purpose of comparing outcomes of children induced at late term with those born post-term by expectant management [57]. Finally, the generalisability of our findings may vary with regional differences in practice regarding the management of pre- or post-term pregnancy and in the quality of obstetric and neonatal care.

Our findings are consistent with other studies examining risk of cognitive deficit in relation to birth before or after term gestational duration [12, 16–19, 58–64]. These studies suggest there may be increased risk of intellectual disability [12, 59], special educational needs [17, 62, 63], poorer performance in school [12, 18, 58, 60, 63] and lower IQ in childhood [16, 64] or adulthood [19, 61] when children are born before or after term. Furthermore, our findings are consistent with those of other studies investigating variation within the term period and reporting that birth at 40–41 weeks’ gestation was optimal in relation to IQ scores at age six [16], risk of special educational needs in primary or secondary school [17], and end-of-secondary school performance outcomes [18]. The independent risk of intellectual disability associated with being born small-for-gestational age is consistent with earlier studies examining other outcomes for fetal growth-restriction in infants born at preterm or post-term gestational duration [16, 58, 65, 66].

In conclusion, our findings suggest that delivery at non-optimal gestational age is associated with greater risk of intellectual disability in offspring in the absence of common genetic causes. This association existed independently of a range of measured potential confounders as well as unmeasured confounding from shared familial factors. While this study cannot provide conclusive evidence for causality, our use of a matched sibling design helped to address confounding due to unmeasured shared familial factors, thereby providing a better estimate of the causal effect than in studies using traditional methods for dealing with confounding. As birth at non-optimal gestational duration may be linked causally with greater risk of intellectual disability, it is important that the mechanisms underlying these associations are elucidated because of their relevance to clinical practice concerning elective delivery within the term period and the mitigation of risk in children who are born post-term. Our work highlights the need for randomized controlled studies to establish whether offspring neurodevelopmental outcomes would improve if women in post-term pregnancies were routinely induced.

## Electronic supplementary material

Below is the link to the electronic supplementary material.

Supplementary material 1 (DOCX 108 kb)

## References

[1] American Psychiatric Association. Diagnostic and statistical manual of mental disorders: DSM-IV. Washington DC: American Psychiatric Association (APA); 1994.

[2] World Health Organisation. Definition: intellectual disability. WHO. 2016. http://www.euro.who.int/en/health-topics/noncommunicable-diseases/mental-health/news/news/2010/15/childrens-right-to-family-life/definition-intellectual-disability. Accessed 05 May 2016.

[3] King BH, Toth KE, Hodapp RM, Dykens EM, Sadock BJSV, Ruiz P (2009). Intellectual disability. Comprehensive textbook of psychiatry.

[4] Lakhan R, Ekundayo OT, Shahbazi M (2015). An estimation of the prevalence of intellectual disabilities and its association with age in rural and urban populations in India. J Neurosci Rural Pract.

[5] Maulik PK, Mascarenhas MN, Mathers CD, Dua T, Saxena S (2011). Prevalence of intellectual disability: a meta-analysis of population-based studies. Res Dev Disabil.

[6] McKenzie K, Milton M, Smith G, Ouellette-Kuntz H (2016). Systematic review of the prevalence and incidence of intellectual disabilities: current trends and issues. Curr Dev Disord Rep.

[7] Doran CM, Einfeld SL, Madden RH (2012). How much does intellectual disability really cost? First estimates for Australia. J Intellect Dev Disabil.

[8] Ali A, Hassiotis A, Strydom A, King M (2012). Self stigma in people with intellectual disabilities and courtesy stigma in family carers: a systematic review. Res Dev Disabil.

[9] Heslop P, Blair P, Fleming P, Hoghton M, Marriott A, Rull L (2013). Confidential inquiry into premature deaths of people with learning disabilities (CIPOLD).

[10] Shapiro BK, Batshaw ML, Kliegman RM, Behrman RE, Jenson HB, Stanton BF (2011). Intellectual disability. Nelson textbook of pediatrics.

[11] Huang J, Zhu T, Qu Y, Mu D (2016). Prenatal, perinatal and neonatal risk factors for intellectual disability: a systemic review and meta-analysis. PLoS ONE.

[12] Moster D, Lie RT, Markestad T (2008). Long-term medical and social consequences of preterm birth. N Engl J Med.

[13] Shime J, Librach CL, Gare DJ, Cook CJ (1986). The influence of prolonged pregnancy on infant development at one and two years of age: a prospective controlled study. Am J Obstet Gynecol.

[14] Lindstrom K, Fernell E, Westgren M (2005). Developmental data in preschool children born after prolonged pregnancy. Acta Paediatr.

[15] El Marroun H, Zeegers M, Steegers EAP (2012). Post-term birth and the risk of behavioural and emotional problems in early childhood. Int J Epidemiol.

[16] Yang S, Platt RW, Kramer MS (2010). Variation in child cognitive ability by week of gestation among healthy term births. Am J Epidemiol.

[17] MacKay DF, Smith GC, Dobbie R, Pell JP (2010). Gestational age at delivery and special educational need: retrospective cohort study of 407,503 schoolchildren. PLoS Med.

[18] Abel KM, Heuvelman H, Wicks S (2016). Gestational age at birth and academic performance: population-based cohort study. Int J Epidemiol.

[19] Eide MG, Oyen N, Skjaerven R, Bjerkedal T (2007). Associations of birth size, gestational age, and adult size with intellectual performance: evidence from a cohort of Norwegian men. Pediatr Res.

[20] Terry AR, Barker FG, Leffert L, Bateman BT, Souter I, Plotkin SR (2013). Neurofibromatosis type 1 and pregnancy complications: a population-based study. Am J Obstet Gynecol.

[21] Kase JS, Visintainer P (2007). The relationship between congenital malformations and preterm birth. J Perinat Med.

[22] Idring S, Rai D, Dal H (2012). Autism spectrum disorders in the Stockholm Youth Cohort: design, Prevalence and Validity. PLoS ONE.

[23] Zeitlin J, Saurel-Cubizolles M-J, de Mouzon J (2002). Fetal sex and preterm birth: are males at greater risk?. Hum Reprod.

[24] Schempf AH, Branum AM, Lukacs SL, Schoendorf KC (2007). Maternal age and parity-associated risks of preterm birth: differences by race/ethnicity. Paediatr Perinat Epidemiol.

[25] Xiong X, Saunders LD, Wang FL, Demianczuk NN (2001). Gestational diabetes mellitus: prevalence, risk factors, maternal and infant outcomes. Int J Gynecol Obstet.

[26] Ananth CV, Savitz DA, Luther ER, Bowes WA (1997). Preeclampsia and preterm birth subtypes in Nova Scotia, 1986 to 1992. Am J Perinatol.

[27] Li X, Sundquist J, Sundquist K (2013). Immigrants and preterm births: a nationwide epidemiological study in Sweden. Matern Child Health J.

[28] Morgan VA, Croft ML, Valuri GM (2012). Intellectual disability and other neuropsychiatric outcomes in high-risk children of mothers with schizophrenia, bipolar disorder and unipolar major depression. Br J Psychiatry.

[29] Mannisto T, Mendola P, Kiely M (2016). Maternal psychiatric disorders and risk of preterm birth. Ann Epidemiol.

[30] Li X, Sundquist J, Kane K, Jin Q, Sundquist K (2010). Parental occupation and preterm births: a nationwide epidemiological study in Sweden. Paediatr Perinat Epidemiol.

[31] Zheng X, Chen R, Li N (2011). Socioeconomic status and children with intellectual disability in China. J Intellect Disabil Res.

[32] Ruiz M, Goldblatt P, Morrison J (2015). Mother’s education and the risk of preterm and small for gestational age birth: a DRIVERS meta-analysis of 12 European cohorts. J Epidemiol Community Health.

[33] Stata. xtgee—fit population-averaged panel-data models by using GEE. Stata. http://www.stata.com/manuals13/xtxtgee.pdf. Accessed 10 Oct 2016.

[34] Stata. mkspline—linear and restricted cubic spline construction. Stata. http://www.stata.com/manuals13/rmkspline.pdf. Accessed 10 Oct 2016.

[35] Lahey BB, D’Onofrio BM (2010). All in the family: comparing siblings to test causal hypotheses regarding environmental influences on behavior. Curr Dir Psychol Sci.

[36] Shea V, Mesibov G, Volkmar FR, Paul R, Klin A, Cohen D (2005). Adolescents and adults with autism. Handbook of autism and pervasive developmental disorders.

[37] Chakrabarti S, Fombonne E (2005). Pervasive developmental disorders in preschool children: confirmation of high prevalence. Am J Psychiatry.

[38] Voigt RG, Barbaresi WJ, Colligan RC, Weaver AL, Katusic SK (2006). Developmental dissociation, deviance, and delay: occurrence of attention-deficit-hyperactivity disorder in individuals with and without borderline-to-mild intellectual disability. Dev Med Child Neurol.

[39] Leavey A, Zwaigenbaum L, Heavner K, Burstyn I (2013). Gestational age at birth and risk of autism spectrum disorders in Alberta, Canada. J Pediatr.

[40] Sucksdorff M, Lehtonen L, Chudal R (2015). Preterm birth and poor Fetal growth as risk factors of attention-deficit/hyperactivity disorder. Pediatrics.

[41] Kaufman L, Ayub M, Vincent JB (2010). The genetic basis of non-syndromic intellectual disability: a review. J Neurodev Disord.

[42] Lie RT, Wilcox AJ, Skjaerven R (2006). Maternal and paternal influences on length of pregnancy. Obstet Gynecol.

[43] Haglund B (2007). Birthweight distributions by gestational age: comparison of LMP-based and ultrasound-based estimates of gestational age using data from the Swedish Birth Registry. Paediatr Perinat Epidemiol.

[44] Dietz PM, England LJ, Callaghan WM, Pearl M, Wier ML, Kharrazi M (2007). A comparison of LMP-based and ultrasound-based estimates of gestational age using linked California livebirth and prenatal screening records. Paediatr Perinat Epidemiol.

[45] Hoffman CS, Messer LC, Mendola P, Savitz DA, Herring AH, Hartmann KE (2008). Comparison of gestational age at birth based on last menstrual period and ultrasound during the first trimester. Paediatr Perinat Epidemiol.

[46] Thomas D, Stram A, Dwyer J (1993). Exposure measurement error: influence on exposure-disease relationships and methods of correction. Annu Rev Public Health.

[47] Frisell T, Oberg S, Kuja-Halkola R, Sjolander A (2012). Sibling comparison designs: bias from non-shared confounders and measurement error. Epidemiology.

[48] Dammann O, Kuban KC, Leviton A (2002). Perinatal infection, fetal inflammatory response, white matter damage, and cognitive limitations in children born preterm. Mental Retard Dev Disabil Res Rev.

[49] Sharashova EE, Anda EE, Grjibovski AM (2014). Early pregnancy body mass index and spontaneous preterm birth in Northwest Russia: a registry-based study. BMC Pregnancy Childbirth.

[50] Basatemur E, Gardiner J, Williams C, Melhuish E, Barnes J, Sutcliffe A (2013). Maternal prepregnancy BMI and child cognition: a longitudinal cohort study. Pediatrics.

[51] Forray A (2016). Substance use during pregnancy. F1000Research.

[52] Ortinau C, Neil J (2015). The neuroanatomy of prematurity: normal brain development and the impact of preterm birth. Clin Anat.

[53] Pavlova MA, Krageloh-Mann I (2013). Limitations on the developing preterm brain: impact of periventricular white matter lesions on brain connectivity and cognition. Brain.

[54] Polglase GR, Miller SL, Barton SK (2014). Respiratory support for premature neonates in the delivery room: effects on cardiovascular function and the development of brain injury. Pediatr Res.

[55] Galal M, Symonds I, Murray H, Petraglia F, Smith R (2012). Postterm pregnancy. Facts Views Vis ObGyn.

[56] Gulmezoglu AM, Crowther CA, Middleton P, Heatley E (2012). Induction of labour for improving birth outcomes for women at or beyond term. Cochrane Database Syst Rev.

[57] Elden H, Hagberg H, Wessberg A (2016). Study protocol of SWEPIS a Swedish multicentre register based randomised controlled trial to compare induction of labour at 41 completed gestational weeks versus expectant management and induction at 42 completed gestational weeks. BMC Pregnancy Childbirth.

[58] Noble KG, Fifer WP, Rauh VA, Nomura Y, Andrews HF (2012). Academic achievement varies with gestational age among children born at term. Pediatrics.

[59] Larroque B, Ancel PY, Marret S (2008). Neurodevelopmental disabilities and special care of 5-year-old children born before 33 weeks of gestation (the EPIPAGE study): a longitudinal cohort study. Lancet.

[60] Lipkind HS, Slopen ME, Pfeiffer MR, McVeigh KH (2012). School-age outcomes of late preterm infants in New York City. Am J Obstet Gynecol.

[61] Ekeus C, Lindstrom K, Lindblad F, Rasmussen F, Hjern A (2010). Preterm birth, social disadvantage, and cognitive competence in Swedish 18- to 19-year-old men. Pediatrics.

[62] Morse SB, Zheng H, Tang Y, Roth J (2009). Early school-age outcomes of late preterm infants. Pediatrics.

[63] Chyi LJ, Lee HC, Hintz SR, Gould JB, Sutcliffe TL (2008). School outcomes of late preterm infants: special needs and challenges for infants born at 32 to 36 weeks gestation. J Pediatr.

[64] Talge NM, Holzman C, Wang J, Lucia V, Gardiner J, Breslau N (2010). Late-preterm birth and its association with cognitive and socioemotional outcomes at 6 years of age. Pediatrics.

[65] Morken NH, Klungsoyr K, Skjaerven R (2014). Perinatal mortality by gestational week and size at birth in singleton pregnancies at and beyond term: a nationwide population-based cohort study. BMC Pregnancy Childbirth.

[66] Abel KM, Dalman C, Svensson AC (2013). Deviance in fetal growth and risk of autism spectrum disorder. Am J Psychiatry.

