# Supplementary material for: Gestational age at birth and risk of intellectual disability without a common genetic cause
Source: Eur J Epidemiol. 2017 Dec 6;33(7):667–78. doi: 10.1007/s10654-017-0340-1 (PMC6061122; doi:10.1007/s10654-017-0340-1)
Supplement: Supplementary file 1 — Supplementary material 1 (DOCX 108 kb) [file 10654_2017_340_MOESM1_ESM.docx]

Table S1. Excluded genetic and inborn metabolic syndromes

|  | **ICD-10** | | **ICD-9** | | **ICD-8** | |
| --- | --- | --- | --- | --- | --- | --- |
|  | **diagnostic code and description** | | **diagnostic code and description** | | **diagnostic code and description** | |
| **Genetic defects** | Q85.0 | Neurofibromatosis (non-malignant) | 237.7 | Neurofibromatosis  (uncertain behavior) | 743.4 | Neurofibromatosis |
|  | Q85.1 | Tuberous sclerosis | 759.5 | Tuberous sclerosis | 759.6 | Tuberous sclerosis |
|  | All Q90-Q99 | Chromosomal abnormalities, not elsewhere specified | 758 | Chromosomal anomalies | 759.3 | Down's syndrome |
|  |  |  |  |  | 759.4 | Other syndromes due to autosomal abnormality |
|  |  |  |  |  | 759.5 | Syndromes due to sex chromosome abnormality |
|  |  |  |  |  | 759.8 | Other specified syndromes |
|  |  |  |  |  | 759.9 | Multiple congenital anomalies, unspecified |
| **Inborn errors of metabolism** | All of E70-E72 | Metabolic disorders | All of 270 | Disorders of amino-acid transport and metabolism | All of 270 | Congenital disorders of amino-acid metabolism |

Table S2. Characteristics of individuals with complete and missing data

|  | | Complete data ^a^ | Missing data | Association between characteristic and risk of missing data |
| --- | --- | --- | --- | --- |
| Number of observations | | 499,881 | 24,896 |  |
| Percentage of the cohort | | 95.26 | 4.74 |  |
|  | | % | % | OR (95% CI) ^b^ |
| Female | | 48.8 | 48.1 | 0.97 (0.95 – 1.00) |
| Gestational hypertension or preeclampsia | No | 96.4 | 92.4 |  |
|  | Yes | 3.6 | 4.1 | 1.19 (1.12 – 1.27) |
|  | Missing | 0.0 | 3.5 |  |
| Gestational diabetes | No | 99.2 | 95.7 |  |
|  | Yes | 0.8 | 0.7 | 0.92 (0.79 – 1.06) |
|  | Missing | 0.0 | 3.5 |  |
| Maternal psychiatric history | No | 67.1 | 68.0 |  |
|  | Yes | 32.9 | 32.1 | 0.96 (0.94 – 0.99) |
| Paternal psychiatric history | No | 79.1 | 79.9 |  |
|  | Yes | 20.9 | 20.1 | 0.95 (0.92 – 0.98) |
| Mother's country of birth | Sweden | 76.1 | 66.7 |  |
|  | Other | 23.9 | 33.2 | 1.59 (1.55 – 1.63) |
|  | Missing | 0.0 | 0.1 |  |
| Father's country of birth | Sweden | 74.9 | 65.2 |  |
|  | Other | 25.1 | 34.5 | 1.58 (1.54 – 1.62) |
|  | Missing | 0.0 | 0.3 |  |
| Family disposable income quintile around birth | Lowest | 14.6 | 27.1 | 2.07 (1.99 – 2.15) |
|  | Second | 20.6 | 17.1 | 0.93 (0.89 – 0.96) |
|  | Third | 21.5 | 16.3 | 0.85 (0.81 – 0.88) |
|  | Fourth | 21.7 | 16.5 | 0.85 (0.81 – 0.88) |
|  | Highest | 21.6 | 19.3 |  |
|  | Missing | 0.0 | 3.7 |  |
| Highest parental educational attainment around birth | ≤9 years | 6.6 | 6.6 | 0.94 (0.89 – 0.99) |
|  | 10 - 11 years | 40.6 | 31.4 | 0.72 (0.70 – 0.74) |
|  | ≥13 years | 52.8 | 56.8 |  |
|  | Missing | 0.0 | 5.2 |  |
| Maternal age | <20 | 1.8 | 1.9 | 1.18 (1.07 – 1.30) |
|  | 20-24 | 14.6 | 13.1 |  |
|  | 25-29 | 31.0 | 28.6 | 1.08 (1.02 – 1.14) |
|  | 30-34 | 33.7 | 35.4 | 1.22 (1.15 – 1.29) |
|  | 35-39 | 15.7 | 17.3 | 1.32 (1.24 – 1.40) |
|  | 40+ | 3.2 | 3.6 | 1.44 (1.36 – 1.53) |
| Paternal age | <20 | 0.5 | 0.5 | 1.19 (0.99 – 1.44) |
|  | 20-24 | 7.3 | 6.0 |  |
|  | 25-29 | 23.5 | 20.7 | 1.08 (1.02 – 1.14) |
|  | 30-34 | 33.4 | 33.3 | 1.22 (1.15 – 1.29) |
|  | 35-39 | 21.9 | 23.6 | 1.32 (1.24 – 1.40) |
|  | 40+ | 13.4 | 15.8 | 1.44 (1.36 – 1.53) |
|  | Missing | 0.0 | 0.2 |  |
| Intellectual disability | | 1.0 | 1.6 | 1.57 (1.42 – 1.74) |

Notes: (a) Individuals has complete data on gestational age and covariate measures. (b) Odds ratio with 95% confidence interval. (c) Assuming the associations reported in Table 1 in the main text, higher prevalence of parental foreign-birth and advanced parental age suggests prevalence of preterm birth may have been higher among those who were excluded due to having missing data.

Fig S1. Population-level associations between risk of intellectual disability and gestational duration among those without ASD or ADHD.


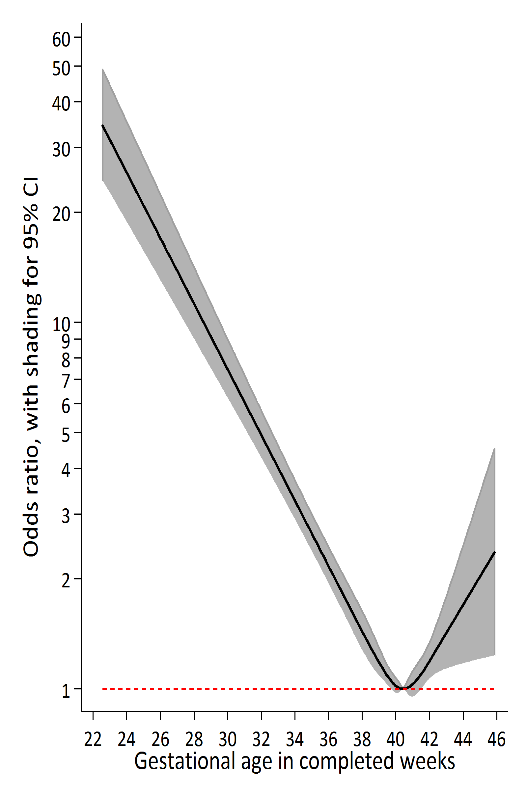


Notes: The population-level association between gestational duration and risk of intellectual disability (N=476,957) was estimated using a generalized estimating equations model with a logit link, and adjusted statistically for year of birth, child sex, parity, gestational hypertension or preeclampsia, gestational diabetes, birth weight for gestational age, maternal and paternal age, maternal and paternal psychiatric history, maternal and paternal country of birth, family disposable income quintile at birth, and parental educational attainment at birth. Those born at 40 weeks and 3 gestational days are the referent.

Table S3. Population-level associations between risk of intellectual disability and gestational duration among those without ASD or ADHD.

| Number of completed weeks | Odds ratio | 95% CI | | *p* | n ^a^ | N ^b^ |
| --- | --- | --- | --- | --- | --- | --- |
|  |  | (lower | upper) |  |  |  |
| 21 - 31 | 8.25 | (6.49 | 10.48) | <0.001 | 79 | 2,323 |
| 32 - 36 | 2.22 | (1.90 | 2.60) | <0.001 | 185 | 19,073 |
| 37 - 41 | 1.00 ^c^ |  |  |  | 1,694 | 418,827 |
| 42 | 1.07 | (0.90 | 1.27) | 0.44 | 139 | 33,227 |
| 43 - 45 | 1.68 | (1.13 | 2.49) | 0.011 | 25 | 3,507 |

Notes: (a) Number of ID cases within gestational age category; (b) Number of observations within gestational age category. (c) Those born between 37 and 41 completed weeks are the referent. (d) Population-level associations (N=476,957) were estimated using a generalized estimating equations model with a logit link, and adjusted statistically for year of birth, child sex, parity, gestational hypertension or preeclampsia, gestational diabetes, birth weight for gestational age, maternal and paternal age, maternal and paternal psychiatric history, maternal and paternal country of birth, family disposable income quintile at birth, and parental educational attainment at birth.

Table S4. Risk of intellectual disability among those born at varying gestational duration in vaginal or Caesarean deliveries

|  | | Odds ratio | 95% CI | | *p* | n ^a^ | N ^b^ |
| --- | --- | --- | --- | --- | --- | --- | --- |
|  |  |  | (lower | upper) |  |  |  |
| 21 - 31 weeks | Vaginal | 8.02 | (6.29 | 10.23) | <0.001 | 77 | 1,070 |
|  | Caesarean | 4.65 | (3.63 | 5.96) | <0.001 | 69 | 1,530 |
| 32 - 36 weeks | Vaginal | 1.68 | (1.46 | 1.93) | <0.001 | 221 | 13,699 |
|  | Caesarean | 2.17 | (1.82 | 2.58) | <0.001 | 147 | 6,572 |
| 37 - 41 weeks | Vaginal | 1.00 ^c^ |  |  |  | 3,520 | 380,114 |
|  | Caesarean | 1.23 | (1.12 | 1.35) | <0.001 | 621 | 58,101 |
| 42 weeks | Vaginal | 1.09 | (0.96 | 1.22) | 0.18 | 294 | 29,077 |
|  | Caesarean | 1.36 | (1.06 | 1.76) | 0.017 | 62 | 5,751 |
| 43 - 45 weeks | Vaginal | 1.52 | (1.13 | 2.06) | 0.006 | 44 | 2,873 |
|  | Caesarean | 1.93 | (1.13 | 3.31) | 0.017 | 14 | 834 |

Notes: (a) Number of ID cases within gestational age category; (b) Number of observations within gestational age category. (c) Those born vaginally between 37 and 41 completed weeks are the referent. (d) Population-level associations (N=499,621) were estimated using a generalized estimating equations model with a logit link, and adjusted statistically for year of birth, child sex, parity, gestational hypertension or preeclampsia, gestational diabetes, birth weight for gestational age, maternal and paternal age, maternal and paternal psychiatric history, maternal and paternal country of birth, family disposable income quintile at birth, and parental educational attainment at birth.

Table S5. Risk of intellectual disability among those born at varying gestational duration in unassisted or assisted deliveries

|  | | Odds ratio | 95% CI | | *p* | n ^a^ | N ^b^ |
| --- | --- | --- | --- | --- | --- | --- | --- |
|  |  |  | (lower | upper) |  |  |  |
| 21 - 31 weeks | Unassisted | 5.81 | (4.86 | 6.93) | <0.001 | 144 | 2,564 |
|  | Forceps or ventouse | 6.89 | (1.66 | 28.51) | 0.008 | 2 | 36 |
| 32 - 36 weeks | Unassisted | 1.80 | (1.61 | 2.02) | <0.001 | 354 | 19,321 |
|  | Forceps or ventouse | 1.80 | (1.04 | 3.10) | 0.036 | 14 | 950 |
| 37 - 41 weeks | Unassisted | 1.00 ^c^ |  |  |  | 3,814 | 403,120 |
|  | Forceps or ventouse | 1.12 | (0.99 | 1.25) | 0.065 | 327 | 35,095 |
| 42 weeks | Unassisted | 1.13 | (1.00 | 1.26) | 0.045 | 316 | 29,928 |
|  | Forceps or ventouse | 0.96 | (0.70 | 1.33) | 0.82 | 40 | 4,900 |
| 43 - 45 weeks | Unassisted | 1.51 | (1.13 | 2.01) | 0.006 | 48 | 3,154 |
|  | Forceps or ventouse | 2.03 | (1.08 | 3.81) | 0.027 | 10 | 553 |

Notes: (a) Number of ID cases within gestational age category; (b) Number of observations within gestational age category. (c) Those born between 37 and 41 completed weeks in unassisted deliveries are the referent. (d) Population-level associations (N=499,621) were estimated using a generalized estimating equations model with a logit link, and adjusted statistically for year of birth, child sex, parity, gestational hypertension or preeclampsia, gestational diabetes, birth weight for gestational age, maternal and paternal age, maternal and paternal psychiatric history, maternal and paternal country of birth, family disposable income quintile at birth, and parental educational attainment at birth.

Table S6. Risk of intellectual disability among those born at varying gestational duration in spontaneous or induced deliveries

|  | | Odds ratio | 95% CI | | *p* | n ^a^ | N ^b^ |
| --- | --- | --- | --- | --- | --- | --- | --- |
|  |  |  | (lower | upper) |  |  |  |
| 21 - 31 weeks | Spontaneous | 5.73 | (4.65 | 7.07) | <0.001 | 103 | 1,990 |
|  | Induced | 5.07 | (0.77 | 33.43) | 0.092 | 1 | 21 |
| 32 - 36 weeks | Spontaneous | 1.83 | (1.59 | 2.11) | <0.001 | 225 | 13,108 |
|  | Induced | 1.94 | (1.30 | 2.91) | 0.001 | 26 | 1,456 |
| 37 - 41 weeks | Spontaneous | 1.00 ^c^ |  |  |  | 2,671 | 303,315 |
|  | Induced | 1.26 | (1.10 | 1.45) | <0.001 | 259 | 23,845 |
| 42 weeks | Spontaneous | 1.04 | (0.88 | 1.21) | 0.67 | 163 | 17,032 |
|  | Induced | 1.36 | (1.10 | 1.68) | 0.005 | 90 | 8,593 |
| 43 - 45 weeks | Spontaneous | 1.25 | (0.76 | 2.06) | 0.38 | 16 | 1,204 |
|  | Induced | 1.74 | (1.10 | 2.75) | 0.018 | 19 | 1,241 |

Notes: (a) Number of ID cases within gestational age category; (b) Number of observations within gestational age category. (c) Those born between 37 and 41 completed weeks in spontaneous deliveries are the referent. (d) Population-level associations (N=371,805) were estimated using a generalized estimating equations model with a logit link, and adjusted statistically for year of birth, child sex, parity, gestational hypertension or preeclampsia, gestational diabetes, birth weight for gestational age, maternal and paternal age, maternal and paternal psychiatric history, maternal and paternal country of birth, family disposable income quintile at birth, and parental educational attainment at birth.

Table S7. Population-level and within-family associations between gestational duration and risk of intellectual disability

|  | Population-level association ^a^ | | | | | | Within-family association ^b^ | | | | | |
| --- | --- | --- | --- | --- | --- | --- | --- | --- | --- | --- | --- | --- |
| Number of completed weeks | Odds ratio | 95% CI | |  |  |  | Odds ratio | 95% CI | |  |  |  |
|  |  | (lower | upper) | *p* | n ^c^ | N ^d^ |  | (lower | upper) | *p* | n ^c^ | N ^d^ |
| 21 - 31 | 5.72 | (4.80 | 6.82) | <0.001 | 146 | 2,601 | 7.84 | (4.55 | 13.50) | <0.001 | 85 | 112 |
| 32 - 36 | 1.78 | (1.60 | 2.00) | <0.001 | 368 | 20,271 | 1.79 | (1.42 | 2.24) | <0.001 | 213 | 430 |
| 37 - 41 | 1.00 ^e^ |  | |  | 4,141 | 438,215 | 1.00 ^e^ |  | |  | 2,706 | 6,836 |
| 42 | 1.08 | (0.97 | 1.21) | 0.15 | 356 | 34,828 | 1.21 | (0.99 | 1.48) | 0.056 | 252 | 579 |
| 43 - 45 | 1.54 | (1.19 | 2.01) | 0.001 | 58 | 3,706 | 2.07 | (1.28 | 3.36) | 0.003 | 40 | 77 |

Notes: (a) Population-level associations (N=499,621) were estimated using a generalized estimating equations model with a logit link, and adjusted statistically for year of birth, child sex, parity, gestational hypertension or preeclampsia, gestational diabetes, birth weight for gestational age, maternal and paternal age, maternal and paternal psychiatric history, maternal and paternal country of birth, family disposable income quintile at birth, and parental educational attainment at birth. (b) Within-family associations (N=8,034) were estimated using a conditional likelihood logistic regression model, and adjusted statistically for year of birth, child sex, parity, gestational hypertension or preeclampsia, gestational diabetes, birth weight for gestational age, maternal and paternal age, family disposable income quintile at birth, and parental educational attainment at birth. (c) Number of ID cases within gestational age category; (d) Number of observations within gestational age category; (e) Those born between 37 and 41 completed weeks are the referent.

Table S8. Exclusions due to improbable combination of weight and gestational age

|  |  | Improbable combinations of birth weight and gestational age ^a^ | |
| --- | --- | --- | --- |
| Gestational age in completed weeks | N | n | % within gestational age category |
| 22 | 1 | 0 | 0.00 |
| 23 | 20 | 1 | 5.00 |
| 24 | 66 | 2 | 3.03 |
| 25 | 126 | 5 | 3.97 |
| 26 | 192 | 2 | 1.04 |
| 27 | 262 | 6 | 2.29 |
| 28 | 285 | 3 | 1.05 |
| 29 | 403 | 1 | 0.25 |
| 30 | 532 | 7 | 1.32 |
| 31 | 750 | 9 | 1.20 |
| 32 | 1,042 | 9 | 0.86 |
| 33 | 1,712 | 7 | 0.41 |
| 34 | 2,744 | 7 | 0.26 |
| 35 | 4,957 | 8 | 0.16 |
| 36 | 9,862 | 15 | 0.15 |
| 37 | 23,523 | 28 | 0.12 |
| 38 | 68,386 | 35 | 0.05 |
| 39 | 113,559 | 37 | 0.03 |
| 40 | 140,409 | 32 | 0.02 |
| 41 | 92,488 | 18 | 0.02 |
| 42 | 34,837 | 9 | 0.03 |
| 43 | 3,438 | 2 | 0.06 |
| 44 | 221 | 0 | 0.00 |
| 45 | 49 | 0 | 0.00 |
| Total | 499,864 | 243 | 0.05 |

Notes: (a) Improbable combinations of birth weight and gestational age were defined as values more than 3 interquartile ranges below the 25^th^ percentile, or more than 3 interquartile ranges above the 75^th^ percentile, of sex- and week-specific birth weight distributions.
